# Revisiting drug–protein interaction prediction: a novel global–local perspective

**DOI:** 10.1093/bioinformatics/btae271

**Published:** 2024-04-22

**Authors:** Zhecheng Zhou, Qingquan Liao, Jinhang Wei, Linlin Zhuo, Xiaonan Wu, Xiangzheng Fu, Quan Zou

**Affiliations:** School of Data Science and Artificial Intelligence, Wenzhou University of Technology, Wenzhou 325027, China; College of Computer Science and Electronic Engineering, Hunan University, Changsha 410012, China; School of Data Science and Artificial Intelligence, Wenzhou University of Technology, Wenzhou 325027, China; School of Data Science and Artificial Intelligence, Wenzhou University of Technology, Wenzhou 325027, China; School of Data Science and Artificial Intelligence, Wenzhou University of Technology, Wenzhou 325027, China; College of Computer Science and Electronic Engineering, Hunan University, Changsha 410012, China; Institute of Fundamental and Frontier Sciences, University of Electronic Science and Technology of China, Chengdu 611730, China

## Abstract

**Motivation:**

Accurate inference of potential drug–protein interactions (DPIs) aids in understanding drug mechanisms and developing novel treatments. Existing deep learning models, however, struggle with accurate node representation in DPI prediction, limiting their performance.

**Results:**

We propose a new computational framework that integrates global and local features of nodes in the drug–protein bipartite graph for efficient DPI inference. Initially, we employ pre-trained models to acquire fundamental knowledge of drugs and proteins and to determine their initial features. Subsequently, the MinHash and HyperLogLog algorithms are utilized to estimate the similarity and set cardinality between drug and protein subgraphs, serving as their local features. Then, an energy-constrained diffusion mechanism is integrated into the transformer architecture, capturing interdependencies between nodes in the drug–protein bipartite graph and extracting their global features. Finally, we fuse the local and global features of nodes and employ multilayer perceptrons to predict the likelihood of potential DPIs. A comprehensive and precise node representation guarantees efficient prediction of unknown DPIs by the model. Various experiments validate the accuracy and reliability of our model, with molecular docking results revealing its capability to identify potential DPIs not present in existing databases. This approach is expected to offer valuable insights for furthering drug repurposing and personalized medicine research.

**Availability and implementation:**

Our code and data are accessible at: https://github.com/ZZCrazy00/DPI.

## 1 Introduction

With the deterioration of the ecological environment, new pathogens and diseases are constantly evolving (Baumgart and Sandborn 2007). This situation is exacerbated by increasing resistance to antibiotics and other drugs, spurring the development of new treatments (Spellberg *et al.* 2008). A major challenge in the early stages of drug development is accurately predicting potential DPIs during the initial screening of viable compounds (Burrows *et al.* 2011). Furthermore, accurate DPI prediction not only sheds light on drug mechanisms but also aids in designing new treatment options, thus, adhering to the principles of safe and precise medication. However, the complexity of biological systems and the vast array of potential compounds make efficiently completing DPI prediction tasks quite challenging (Butcher *et al.* 2004). In response to these challenges, researchers are advancing computational methods to more accurately simulate and predict DPIs (Li *et al.* 2022, Sun *et al.* 2023).

Molecular docking, a common technique in drug design and discovery, simulates the binding process between drug molecules and protein targets (Wang *et al.* 2023b). It involves matching a drug molecule with a target protein’s binding pocket through numerous spatial orientations and conformations. Typically, each binding approach utilizes a unique set of scoring functions to assess the likelihood of binding. These scoring functions quantify the binding stability between drug molecules and protein targets, predicting their affinity. Despite its power, molecular docking’s high computational complexity renders it unsuitable for large-scale, high-throughput screening (Zhang *et al.* 2014).

The emergence of deep learning technology is driving transformative changes in biology (Wang *et al.* 2023a). Deep learning’s efficient data processing has led to breakthroughs in protein structure prediction, gene expression analysis, and disease diagnosis (Wang *et al.* 2023b). Inspired by this, researchers have developed computational models for rapidly screening drugs with high affinity to specific protein targets (Wei *et al.* 2023). Prominent deep learning technologies primarily encompass graph neural networks (GNN), convolutional neural networks (CNN), and transformers. GNN-based methods are adept at uncovering complex molecular topologies, ideal for representing compounds. Originally developed for computer vision and natural language processing, CNNs and transformers have been effectively adapted to extract protein sequence features. The model learns node representations from DPI data to predict potential molecular binding properties. This enables the guidance of drug design and synthesis, accelerating new drug discovery and development.

Hakime *et al.* introduced DeepDTA, a deep learning model, for predicting DPIs. This model employs CNN technology to extract sequence features of proteins and drugs, followed by multilayer perceptron (MLP) for inferring DPI probabilities (Öztürk *et al.* 2018). Utilizing GNN technology, Nguyen *et al.* represented drug molecules with graphs and applied GNN models to predict drug–target affinity (Nguyen *et al.* 2021). Huang introduced the MolTrans model, leveraging an enhanced transformer encoder to predict potential drug–target interactions (DTIs). A key feature of MolTrans is its ability to model drug–target interactions and explore potential substructures, enhancing the model’s interpretability (Huang *et al.* 2021). Liu *et al.* merged GNN technology with the transformer model, proposing a model named IGT. This approach employs a specialized attention mechanism in a tripartite transformer-based architecture to simulate intermolecular information, significantly enhancing the model’s DPI prediction accuracy (Liu *et al.* 2022). Chen *et al.* developed transformerCPI, a model applying transformer and deconvolution technology for compound–protein interaction (CPI) prediction. Notably, the model employs deconvolution technology during training to identify key interaction regions of protein compounds, enhancing both interpretability and predictive accuracy (Chen *et al.* 2020). Cheng *et al.* integrated node and interactive substructure features, employing MLP to predict potential DPIs. They utilized GNN and CNN techniques to extract drug and protein features, respectively, and then, applied an encoder-decoder architecture for feature extraction of interacting substructures (Cheng *et al.* 2022). Dong *et al.* introduced MMA-DPI, a multimodal DPI prediction model based on transformer and GNN technology. The model enhances the transformer module to extract substructural information and chemical semantic representations of molecular interactions, employs GNN technology for learning node representations of multi-source association networks, and fuses multimodal representations to calculate drug–protein pair scores (Dong *et al.* 2023).

While existing deep learning models efficiently predict potential DPIs, they encounter three key challenges. First, many models rely on the sequence or structural information of drugs and proteins in datasets for node representation learning, risking overfitting, and limited model generalizability (Greener *et al.* 2022). Second, current models often predict unknown DPIs based on observed ones, neglecting the potential influence of indirectly or nonconnected nodes (Vohradsky 2001). Third, in drug–protein bipartite graphs, most models overlook the local relationships between drug and protein subgraphs (Lotfi Shahreza *et al.* 2018). Consequently, we propose a novel DPI prediction model employing large-model pre-training, a global diffusion mechanism, and local subgraph extraction technologies, designed to efficiently and accurately infer potential DPIs. The proposed model acquires general knowledge of drugs and proteins through large-scale pre-training and models known DPIs information from both local and global perspectives. The results validate the model’s efficiency in inferring potential DPIs. Our contributions to this research are summarized as follows:

1. We employ a large-model-based pre-training strategy to learn general knowledge about drugs and proteins, enhancing the model’s node representation generalization.
2. We apply MinHash and HyperLogLog algorithms to estimate similarity and set cardinality between drug and protein subgraphs in the drug–protein graph, mining their local relationships.
3. We incorporate an energy-constrained global diffusion mechanism into the transformer architecture, extracting drug and protein features and exploring potential interdependencies between nodes.
4. We assessed the model across various public datasets and conducted molecular docking and related analyses based on the predictions, demonstrating its efficiency in inferring potential DPIs.

## 2 Materials and methods

### 2.1 Data preparation

In our study, we assessed the performance of both the proposed and comparison models using the BindingDB and Davis databases (Davis *et al.* 2011, Gilson *et al.* 2016). BindingDB, a public database, comprises extensive binding affinity data between small molecule compounds and proteins, sourced from literature and public datasets. Within the BindingDB database, we utilized the TDC tool for binarization and filtering, resulting in 3400 drugs and 886 proteins, encompassing a total of 9166 DPIs. The Davis database encompasses over 80% of the human catalytic protein kinase group, including 68 drugs, 379 proteins, and 7320 DPIs. This data was derived from processing the *K*(*d*) values of wet laboratory tests using the TDC tool. Additionally, we assessed multiple models’ performance on the Yamanishi *et al.* dataset, which includes Enzyme, G-protein-coupled receptor (GPCR), ion channel (IC), and nuclear receptor (NR) (Yamanishi *et al.* 2008). This dataset comprises 1481 drugs, 1480 proteins, and a total of 9880 DPIs. Detailed statistical information of this dataset is presented in Supplementary Table S1.

### 2.2 Model overview

Our research introduces a novel DPI prediction model that utilizes large-scale pre-training, a global diffusion mechanism, and local subgraph extraction to efficiently and precisely infer potential DPIs. The model leverages large-scale pre-training to assimilate general knowledge of drugs and proteins, enhancing its generalizability in node representation. Subsequently, the model processes drug–protein bipartite graph from both local and global viewpoints, examining potential interdependencies between nodes and the local interactions within drug–protein subgraphs. This process enriches the node representation, facilitating the model’s efficient inference of potential DPIs.

**Figure 1.**
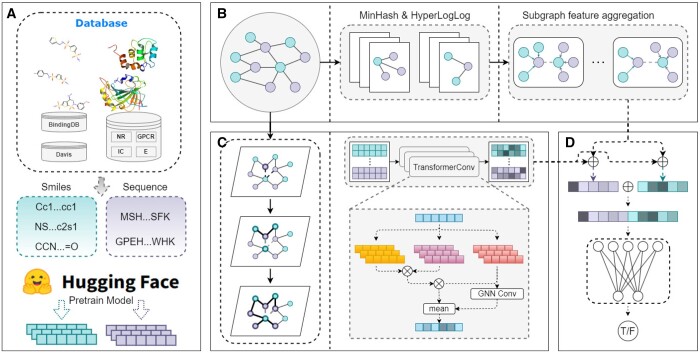
Architectural schematic of the proposed model, comprising four core modules. (A) Retrieve the drug’s SMILES and the protein’s sequence from the database and employ Hugging Face’s pre-trained models to acquire the initial features of the drug and protein. (B) Apply the MinHash and HyperLogLog algorithms within the drug–protein bipartite graph to estimate the similarity and set cardinality between drug and protein subgraphs, serving as their local features. (C) Utilize a transformer encoder, grounded in the global diffusion mechanism, to extract the global features of drugs and proteins. (D) The local and global features derived from modules (B) and (C) are integrated, and a multilayer perceptron is employed to predict the interaction scores of drug–protein pairs.

Figure 1 displays the architectural diagram of the proposed model. The model comprises four main components: (A) the pre-training module, (B) the local subgraph feature extraction module, (C) the global feature extraction module, and (D) the DPI prediction module. In module (A), we initially retrieve the SMILES and protein sequence data for drugs from the BindingDB and Davis databases, along with data from Yamanishi *et al.*’s study. Subsequently, we employ the PubChem10M_SMILES_BPE_450k pre-trained model from Hugging Face to capture the drug’s molecular representation, trained on 10 million chemical molecules from the PubChem database. Concurrently, we utilize the esm2_t33_650M_UR50D pre-trained model from Hugging Face to learn the protein’s molecular representation from its amino acid sequence. This pre-trained model represents the cutting-edge in protein language models that leverage a masking strategy. In module (B), we initially extract k-hop subgraphs for drugs and proteins from the bipartite graph. We apply MinHash and HyperLogLog algorithms to assess the similarity and set cardinality between them, serving as the local features for drug and protein nodes. In module (C), we input the pre-trained features of drugs and proteins into a transformer encoder, which operates on a global diffusion mechanism, to extract their global features. In module (D), we integrate the local and global features derived from modules (B) and (C), and subsequently employ an MLP to predict the interaction score of the drug–protein pair (Supplementary Section 2). Subsequently, we will elucidate the underlying principles and technologies incorporated within these modules.

### 2.3 Pre-trained module

Our study employed two distinct pre-trained models from the Hugging Face library (https://huggingface.co/) to acquire generalized knowledge of drug molecules and proteins. For drug molecules, we initially extracted SMILES strings from the BindingDB database to represent their chemical structures. Subsequently, we employed a pre-trained model, “PubChem10M_SMILES_BPE,” to derive the drugs’ molecular representations from these strings. This model, pre-trained with 10 million SMILES strings from the PubChem database (https://pubchem.ncbi.nlm.nih.gov/), utilizes Byte Pair Encoding (https://huggingface.co/seyonec/PubChem10M_SMILES_BPE_450k) (BPE) to efficiently identify key features of drug molecules, including atomic composition, bond types, and molecular configurations. For proteins, we also extracted the amino acid sequence from the BindingDB database. And the “esm2_t33_650M_UR50D” model is then applied to obtain molecular representations from their amino acid sequences. This model is a large-scale protein language model built on the transformer architecture. Trained on extensive protein sequence data, it can emphasize proteins’ key features, including secondary structures, functional domains, and active sites. These pre-trained models have respectively garnered a comprehensive understanding of drugs and proteins, significantly enhancing the model’s ability to generalize node representations.

### 2.4 Local feature extraction

In the drug–protein bipartite graph, prevailing DPI prediction models often depend on directly connected neighbors for message propagation, which can lead to overfitting. Our study diverges from these models by concentrating on uncovering the local relationships between drug and protein subgraphs. Specifically, we employ a random walk algorithm to extract *k*-hop subgraphs for both drugs and proteins. We then apply MinHash and HyperLogLog algorithms to ascertain the similarity and set cardinality between drug and protein subgraphs, thereby defining their local features (Heule *et al.* 2013, Ondov *et al.* 2016). MinHash provides an efficient estimation of Jaccard similarity between sets, greatly reducing the computational burden associated with direct calculations of Jaccard similarity. The fundamental principle of MinHash involves using a hash function to selectively sample elements from a collection. HyperLogLog employs a probabilistic algorithm to estimate a set’s cardinality, which is defined as the count of unique elements within the set. Its primary objective is to minimize computational overhead. These approaches are anticipated to enrich and refine the node representation of drugs and proteins, thereby enhancing the model’s predictive performance.

### 2.5 Similarity and set cardinality estimation

Initially, each drug (protein) node is centered upon to extract its *k*-hop subgraph using a random walk algorithm. The similarity between drug *d* and protein *p* is expressed as follows:

(1) $$\begin{array}{c} S1_{d,p}[d_{d},d_{p}]=|N_{d_{d},d_{p}}(d,p)|\\ -\sum_{x\leq d_{d},\;y\leq d_{p},\;(x,y)\neq (d_{d},d_{p})}|N_{x,y}(d,p)|, \end{array}$$

where

(2) $$N_{d_{d},d_{p}}(d,p)\equiv N_{d_{d}}(d)\cap N_{d_{p}}(p).$$

The variables $d_{d}$ and $d_{p}$ denote the respective distances to drug *d* and protein *p*, both of which are less than *k*. While $N_{d_{d}}(d)$ and $N_{d_{p}}(p)$ signify the sets of $d_{d}$-order and $d_{p}$-order neighbors for drug *d* and protein *p*, respectively.

For any given drug (or protein *p*) node *d*, nodes outside its fixed subgraph can be crucial. Hence, it’s important to consider higher-order neighbors beyond the predefined k-hop limit.

(3) $$S2_{d,p}[t]=|N_{t}(m)|-S2_{d,p}[t-1]-\sum_{i=1}^{t}\sum_{j=1}^{t}S1_{d,p}[i,j],$$

where $S2_{d,p}[t]$ denotes the count of nodes at a distance *t* from drug *d* and at a distance greater than *k* from protein *p*.

Direct calculation of items $\left|N_{d_{d},d_{p}}(d,p)\right|$ and $\left|N_{t}(d)\right|$ is highly challenging. Employing HyperLogLog and MinHash algorithms for estimation presents a viable solution. Let $a_{d}^{t}$ and $b_{d}^{t}$ denote the *t*-hop neighbors of drug *d* as determined by the HyperLogLog and MinHash methods respectively, with $a_{d}^{t}=max_{p\in N(d)}a_{p}^{t-1}$ and $b_{d}^{t}=min_{p\in N(d)}b_{p}^{t-1}$. Consequently, $\left|N_{d_{d},d_{p}}(d,p)\right|$ can be estimated as follows:

(4) $$\begin{array}{c} \left|N_{d_{d},d_{p}}(d,p)\right|\equiv N_{d_{d}}(d)\cap N_{d_{p}}(p)\\ =J(N_{d_{d}}(d),N_{d_{p}}(p))\cdot |N_{d_{d}}(d)\cup N_{d_{p}}(p)|\\ \approx H(b_{d}^{d_{d}},b_{p}^{d_{p}})\cdot \mathrm{hyper}(\max(a_{d}^{d_{d}},a_{p}^{d_{p}})), \end{array}$$

where J(x,y) denotes the Jaccard similarity between two sets, estimable through $H(x,y)=\frac{1}{n}\sum_{i}^{n}\delta_{x_{i},y_{i}}$, which signifies the Hamming similarity (Norouzi *et al.* 2012) and serves as the MinHash estimation function. And δ denotes the indicator variable, assigned 1 for a match and 0 for a mismatch. While *hyper* denotes the HyperLogLog cardinality estimation function. In Equation (2.4.1), the MinHash algorithm estimates the Jaccard similarity between two sets, while the HyperLogLog algorithm assesses the set’s cardinality. Additionally, $\left|N_{t}(d)\right|$ can be estimated using $\mathrm{hyper}(a_{d}^{t})$.

### 2.6 Extract local features based on GNN

During the message aggregation and updating phase of the GNN encoder, we incorporate the subgraph structural information of the drug (protein) node:

(5) $$e_{d,p}^{l}=\{S2_{d,p}[l],S1_{d,p}[d_{d},l],S1_{d,p}[l,d_{p}]:\forall d_{d},d_{p}\;<\;l\}$$

(6) $$x_{d}^{l}=\varphi^{l}(x_{d}^{l-1},\mathrm{aggregat}e_{p\in N(d)}\tau^{l}(x_{d}^{l-1},x_{p}^{l-1},e_{d,p}^{l})),$$

where φ and τ are trainable linear functions performing internal concatenate operations, $e_{d,p}^{l}$ denotes the embedding of the edge between drug *d* and protein *p* in the *l*-th layer. The term “aggregate” denotes an aggregation function. And $x_{d}^{l}$ and $x_{p}^{l}$ correspond to the node features of drugs and proteins in the *l*-th layer, respectively, while $x^{0}$ signifies the initial features output by the pre-trained models. Additionally, $e_{d,p}$ symbolizes the edge feature, representing the weight of the pair (d,p). As per Equations (5) and (6), message propagation transpires within the subgraph’s local structure. Compared to global message propagation, local message propagation effectively mitigates the issue of over-smoothing. Subsequently, the local features of the drug node *d* and the protein node *p* can be denoted as $d_{\mathrm{local}}$ and $p_{\mathrm{local}}$, respectively.

### 2.7 Global feature extraction

Current DPI prediction models typically infer unknown DPIs from observable ones, neglecting the potential influence of indirectly or nonconnected nodes. Inspired by previous work (Wu *et al.* 2022), this section introduces a transformer architecture that incorporates an energy-constrained diffusion mechanism for extracting global features of drugs and proteins. Within the drug–protein bipartite graph, the diffusion-guided transformer encoder is capable of exploring potential interdependencies between any two nodes, thereby directing message propagation during the node representation update. Subsequently, we will delve into the details of the global diffusion mechanism and the intricately designed transformer.

### 2.8 Transformer based on global diffusion mechanism

Drawing from the preceding analysis in Supplementary Section 1 and Supplementary Equation (2), the diffusion rate between any two nodes can be interpreted as their attention weight. We introduce an attention architecture directed by an energy-constrained global diffusion mechanism. Supplementary Equation (4) delineates the rule for updating node energy, as guided by the global diffusion mechanism. The corresponding transformer architecture, as depicted in Supplementary Fig. S1, can be constructed accordingly.

Supplementary Figure S1 illustrates the operational mode of the transformer architecture steered by the energy-constrained global diffusion mechanism. Initially, the features *X* of drugs or proteins acquired from the pre-trained model are input into the fully connected layer, followed by a normalization operation:

(7) Z=σ(Ω(WX+b)),

where σ denotes the sigmoid activation function, while Ω signifies the layer normalization process. And *W* and *b* represent the trainable matrix and bias of the linear function respectively. Subsequently, the *Q*, *K*, and *V* matrices are derived via linear projection:

(8) $$\begin{array}{c} K^{\left(u,g\right)}=W_{K}^{\left(u,g\right)}Z^{\left(u\right)},\;Q^{\left(u,g\right)}=W_{Q}^{\left(u,g\right)}Z^{\left(u\right)},\\ \begin{array}{c} V^{\left(u,g\right)}=W_{V}^{\left(u,g\right)}Z^{\left(u\right)}, \end{array} \end{array}$$

where $K^{\left(u,g\right)}$, $Q^{\left(u,g\right)}$, and $V^{\left(u,g\right)}$ denote the matrices corresponding to the *g*-th head of the *u*-th layer, respectively. The matrices $W_{K}^{\left(u,g\right)}$, $W_{Q}^{\left(u,g\right)}$, and $W_{V}^{\left(u,g\right)}$ are the trainable weight matrices of the linear layers, while $Z^{\left(u\right)}$ is the embedding matrix for the *u*-th layer.

As indicated in Supplementary Equations (2) and (3), proceed with $L_{2}$ normalization for the *K* and *Q* matrices:

(9) $${\hat{K}}^{\left(u,g\right)}={\left[\frac{K_{i}^{\left(u,g\right)}}{\|K_{i}^{\left(u,g\right)}{\|}_{2}}\right]}_{i=1}^{N},\;{\hat{Q}}^{\left(u,g\right)}={\left[\frac{Q_{i}^{\left(u,g\right)}}{\|Q_{i}^{\left(u,g\right)}{\|}_{2}}\right]}_{i=1}^{N},$$

Ultimately, as depicted in Supplementary Fig. S1, the embedding for layer u + 1 is derived following successive integrations of the *K*, *Q*, and *V* matrices. Subsequently, the global features of the drug node *d* and the protein node *p* can be denoted as $d_{\mathrm{global}}$ and $p_{\mathrm{global}}$, respectively.

## 3 Results

This section presents a thorough analysis of the proposed model, corroborating its efficacy and stability via extensive experimentation. Initially, we detail the model’s pertinent parameters and elucidate the rationale behind their selection to guarantee the experiment’s reproducibility. We then assess the performance of the proposed model against current leading methods across multiple datasets. To delve deeper into the contributions of different model components to overall performance, a series of ablation studies were conducted. Moreover, parameter sensitivity experiments were carried out to determine the model’s responsiveness and adaptability to changes in parameters (Supplementary Section 6).

The models’ performance was evaluated on multiple datasets, including two larger datasets (BindingDB and Davis) and four smaller datasets (Enzyme, IC, GPCR, and NR). For fairness in comparison, a consistent data partitioning strategy was employed across all datasets. In the experiments, the datasets were divided in a 7:1:2 ratio for training, validation, and testing sets, respectively. Hyperparameters were optimized through grid search to secure the most effective parameter configuration for each model. The Adam optimizer was utilized, with a learning rate of 0.001 and a batch size of 512. An early stopping mechanism halts training if the model’s validation performance does not improve significantly for 10 consecutive iterations. Default settings for the local feature extraction module include a subgraph size of 3, an output feature dimension of 64, and a GNN encoder with two layers. For the global feature extraction module, the Transformer is configured with two layers, the attention mechanism with four heads, and the output feature dimension is established at 256.

### 3.1 Performance comparison

Our evaluation spanned across the proposed model and benchmark models, including DeepDTA, GraphDTA, IGT, Moltrans, TransformerCPI, and IIFDTI, across all datasets, with outcomes detailed in Table 1. On the whole, the DeepDTA and GraphDTA models, employing CNN and GNN encoders respectively, exhibited the least favorable results. The MolTrans model, leveraging an enhanced transformer encoder, is adept at extracting semantic correlations between substructures from unlabeled data via interactive modeling and substructure mining, thereby elevating DPI prediction accuracy. Additionally, the IGT model amalgamates GNN with transformer technology to assimilate both topological and spatial molecular information, which sharpens its ability to deduce potential DPIs. The TransformerCPI and IIFDTI models concentrate on accentuating the attributes of drug–protein interaction regions for a more precise prediction of potential DPIs. It is noteworthy that the proposed model attains state-of-the-art performance on nearly all datasets. The proposed model surpasses the second-best IIFDTI models 1.7% and 3.9% in average AUC and AUPR across all datasets. This could be attributed to the proposed model’s pre-training strategy, which integrates global and local node features, enhancing node representation and thus facilitating more efficient DPI prediction. And the comparison of performance under isolation conditions is presented in Supplementary Table S2. This also indicates that the model’s high performance is contingent on precise node representation.

**Table 1.** Comparative performance of all models across datasets.

	Method	BindingDB	Davis	GPCR	IC	NR	Enzyme
AUC	DeepDTA	0.893±0.007	0.889±0.003	0.892±0.007	0.901±0.008	0.854±0.009	0.873±0.007
	GraphDTA	0.901±0.006	0.897±0.004	0.861±0.006	0.876±0.009	0.833±0.005	0.857±0.007
	IGT	0.937±0.021	0.933±0.003	0.944±0.007	0.931±0.009	0.921±0.011	0.927±0.006
	Moltrans	0.925±0.013	0.914±0.004	0.949±0.006	0.943±0.009	0.912±0.011	0.931±0.007
	TransformerCPI	0.955±0.013	0.951±0.002	0.957±0.005	0.944±0.007	0.924±0.009	0.937±0.006
	IIFDTI	0.951±0.005	0.948±0.002	0.952±0.005	0.949±0.007	0.919±0.009	0.940±0.006
	Ours	0.966±0.001	0.984±0.001	0.947±0.009	0.978±0.005	0.935±0.008	0.951±0.001
AUPR	DeepDTA	0.829±0.010	0.820±0.005	0.876±0.011	0.884±0.013	0.831±0.017	0.854±0.015
	GraphDTA	0.825±0.007	0.821±0.003	0.837±0.015	0.853±0.013	0.797±0.017	0.817±0.011
	IGT	0.912±0.011	0.910±0.009	0.923±0.015	0.909±0.013	0.901±0.017	0.914±0.015
	Moltrans	0.877±0.008	0.867±0.008	0.925±0.015	0.911±0.014	0.864±0.019	0.916±0.013
	TransformerCPI	0.919±0.011	0.917±0.002	0.931±0.011	0.915±0.013	0.894±0.015	0.929±0.011
	IIFDTI	0.926±0.005	0.922±0.003	0.926±0.007	0.917±0.009	0.903±0.011	0.923±0.007
	Ours	0.963±0.002	0.978±0.003	0.943±0.007	0.978±0.006	0.934±0.008	0.953±0.001

### 3.2 Ablation experiment

The proposed model encompasses three principal modules: pre-training, global feature extraction, and local feature extraction. A series of ablation studies were conducted to ascertain the contribution of each module to the model’s performance. “w Pretrain” indicates the omission of both global and local feature extraction modules, relying solely on the pre-training module for feature extraction from drugs and proteins. “w/o Global” signifies the exclusion of the global feature extraction module, while “w/o Local” refers to the absence of the local feature extraction module. Ablation experiments were performed on three extensive datasets: BindingDB, Davis, and Enzyme, utilizing metrics such as AUC, AUPR, ACC, SEN, PRE, SPE, *F*1-score, and MCC to assess the results.

Table 2 presents the results of the model’s ablation studies conducted on the BindingDB, Davis, and Enzyme datasets. The model’s performance is notably inferior when it forgoes the global and local feature extraction modules. The removal of either the global or local feature extraction module results in a comparable decline in performance. As indicated in Table 2, model performance declined after the removal of both the local and global feature extraction modules. This demonstrates that both modules contribute to enhancing the model’s performance. Removing the modules for extracting local features of drugs and proteins results in a greater performance decline than removing those for global features. The performance reduction is particularly evident in the *F*1-score and MCC evaluation metrics. In summary, extracting local features of drugs and proteins is more crucial for enhancing model performance.

**Table 2.** Results of ablation experiment.

Datasets	AUC	AUPR	ACC	SEN	PRE	SPE	*F*1-score	MCC
**BingdingDB**								
w Pretrain	0.9463	0.9462	0.8815	0.8026	0.9531	0.9605	0.8714	0.7728
w/o Global	0.9553	0.9509	0.8895	0.8147	0.9580	0.9643	0.8806	0.7879
w/o Local	0.9578	0.9488	0.8671	0.7722	0.9531	0.9620	0.8532	0.7478
Ours	0.9688	0.9654	0.9165	0.9157	0.9171	0.9172	0.9164	0.8330
**Davis**								
w Pretrain	0.9772	0.9667	0.9368	0.9447	0.9301	0.9290	0.9373	0.8737
w/o Global	0.9829	0.9753	0.9532	0.9720	0.9368	0.9344	0.9541	0.9071
w/o Local	0.9801	0.9704	0.9348	0.9214	0.9467	0.9481	0.9339	0.8698
Ours	0.9893	0.9865	0.9501	0.9419	0.9576	0.9583	0.9497	0.9004
**Enzyme**								
w Pretrain	0.9278	0.9208	0.8752	0.8393	0.9042	0.9111	0.8706	0.7524
w/o Global	0.9480	0.9534	0.8846	0.8547	0.9091	0.9145	0.8811	0.7706
w/o Local	0.9319	0.9174	0.8718	0.9111	0.8447	0.8325	0.8766	0.7459
Ours	0.9506	0.9525	0.8846	0.8359	0.9261	0.9333	0.8787	0.7729

### 3.3 Discovering new DPIs

According to the preceding analysis, the model demonstrates efficiency in inferring potential DPIs. Furthermore, it remains to be seen whether the model can identify unvalidated DPIs within the database, a significant challenge for numerous models. To address this, molecular docking technology was utilized to test the DPI predictions made by the model and to assess interactions between relevant proteins and drugs at the molecular level. Molecular docking serves as a crucial technique for probing potential interactions between drug molecules and proteins, and it is instrumental in predicting potential binding sites. Our objective is to uncover protein–drug pairs within the database that, despite being unvalidated, are predicted by the model to have a high likelihood of interaction. In this study, three rounds of molecular docking procedures were executed, as represented in Supplementary Fig. S4. These results validate the accuracy and reliability of our model and suggest its capability to identify and explore unknown drug targets, offering novel insights in drug research and development.

## 4 Conclusion

Investigating potential DPIs is essential in drug discovery and biomedical research. Consequently, there is an urgent need to develop an efficient model for uncovering more unknown DPIs. Our research introduces a model, employing a large-scale pre-training approach to acquire general knowledge of drugs and proteins and extracting node features both globally and locally to efficiently and reliably identify potential DPIs. A series of comparative and parameter-based experiments were conducted, confirming the superiority of our model over others, validating the significance of its key modules, and offering guidance on parameter configurations. We anticipate that this work will deepen the understanding of DPI mechanisms and offer novel insights for future drug design and development.

The proposed model encounters several predictable challenges. Firstly, the model overlooks the multimodal information of drugs and proteins. Specifically, for drugs, it only extracts SMILES features, and for proteins, only amino acid sequence features are extracted. Secondly, the model lacks emphasis on interpretability. In future studies, efforts will be focused on addressing these challenges. We aim to utilize multimodal technology to merge information from various sources, including the structure, sequence, and images of drugs and proteins. This approach is intended to enhance the quality of node representation. Subsequently, we aim to identify key substructures facilitating DPIs, thereby augmenting the model’s interpretability. With these enhancements, the proposed model is anticipated to serve as a valuable resource for endeavors like drug screening and repositioning.

## Supplementary Material

btae271_Supplementary_Data
