# Supplementary material for: Revisiting drug–protein interaction prediction: a novel global–local perspective
Source: Bioinformatics. 2024 Apr 22;40(5):btae271. doi: 10.1093/bioinformatics/btae271 (PMC11087820; doi:10.1093/bioinformatics/btae271)
Supplement: btae271_Supplementary_Data [file btae271_supplementary_data.pdf]

1 Energy-constrained Diffusion Mechanism

The transformer encoder, a central module of our proposed model, is designed based on an attention mechanism guided by an energy-constrained diffusion process. In this section, both drugs and proteins within the drug-protein bipartite graph are referred to as nodes. To elucidate the foundational concept behind the transformer architecture design, we present the energy-constrained diffusion process equation:

$$
\begin{aligned}
& z_a^{h+1} = (1 - \lambda \sum_{b=1}^{N} S_{ab}^h) z_a^h + \lambda \sum_{b=1}^{N} S_{ab}^h z_b^h, \\
& s.t. \quad z_a^0 = x_a,\ E(Z^{h+1}, h+1, \zeta) \leq E(Z^h, h, \zeta),\ h \geq 1, \\
& E(Z^h, h, \zeta) = \|Z - Z^h\| + \alpha \sum_{a,b} \zeta(\|z_a - z_b\|_2^2).
\end{aligned}
\tag{1}
$$

In Equation 1, z_a^{h+1} represents the energy state of node a at time $h+1$, while S_{ab}, defined as the diffusion rate, reflects the rate of energy propagation. The coefficients λ and α serves as modulating both the node's own energy variation and the energy transmitted to other nodes. And the symbol ζ signifies the energy flow function. The first of Equation 1 denotes the energy update equation for node a, where the first term represents node a's energy change, and the second term sums the energy propagated to other nodes. The second of Equation 1 outlines the constraints to ensure energy decline as the system stabilizes. The third of Equation 1 is presented as an energy function, encapsulating the node's own energy change and the potential consistency of energy propagation between any two nodes in the system.

It is challenging to determine a diffusivity value that meets the constraints and satisfies the conditions. Given that λ ranges between (0,1), to ensure the self-energy change in the first of Equation 1 remains non-negative, we can estimate the diffusion rate between nodes a and b as:

$$
\hat{S}_{ab}^h = \frac{f(\|z_a^h - z_b^h\|_2^2)}{\sum_{c=1}^{N} f(\|z_a^h - z_c^h\|_2^2)},
\tag{2}
$$

Here, the diffusion rate $\hat{S}_{ab}$ can be interpreted as the attention weight between nodes a and b. In Equation 2, f is defined as an index for measuring similarity. To guarantee f's value remains non-negative, set $\hat{z}_a^h = \frac{z_a^h}{\|z_a^h\|_2}$ and $\hat{z}_b^h = \frac{z_b^h}{\|z_b^h\|_2}$. Opt for the linear function $f(x) = 2 - 1/2x$. Consequently, f can be further expressed as:

$$
f(\|\hat{z}_a^h - \hat{z}_b^h\|_2^2) = 1 + (\frac{z_a^h}{\|z_a^h\|_2})^\top (\frac{z_b^h}{\|z_b^h\|_2})
\tag{3}
$$

Then, $f(\|\hat{z}_a^h - \hat{z}_b^h\|_2^2)$ is incorporated into the update equation for node a:

$$
\begin{aligned}
\sum_{b=1}^{N} S_{ab}^h z_b^h &= \sum_{b=1}^{N} \frac{1 + (\hat{z}_a^h)^\top \hat{z}_b^h}{\sum_{c=1}^{N} (1 + (\hat{z}_a^h)^\top \hat{z}_c^h)} z_a^h \\
&= \frac{\sum_{b=1}^{N} z_b^h + \left(\sum_{b=1}^{N} \hat{z}_b^h \cdot (z_b^h)^\top\right) \cdot z_a^h}{N + (\hat{z}_a^h)^\top \sum_{c=1}^{N} \hat{z}_c^h}.
\end{aligned}
\tag{4}
$$

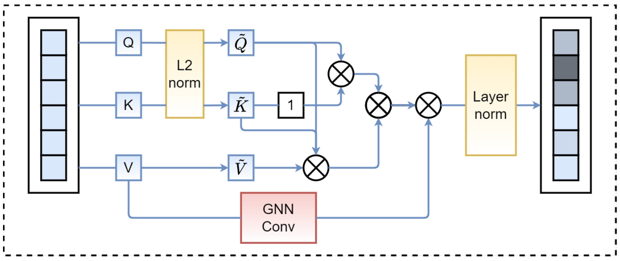

Figure S1: Transformer architecture based on energy-constrained global diffusion mechanism.

2 Model Optimization

Following processing by the local feature extraction (B) and global feature extraction (C) modules, the local and global features of drugs and proteins are computed. For a given drug d and a protein p, their local and

global features of drugs and proteins are then concatenated to form their final representations:

$$X_d = \langle d_{local}, d_{global} \rangle \, , \; X_p = \langle p_{local}, p_{global} \rangle \, . \tag{5}$$

Subsequently, a concatenation process yields the final representation of the drug-protein pair:

$$X_{d,p} = \langle X_d, X_p \rangle \, . \tag{6}$$

Promptly, $X_{d,p}$ is fed into the MLPs for predicting the interaction likelihood between drug d and protein p:

$$\widehat{y}_{d,p} = MLP(X_{d,p}). \tag{7}$$

Ultimately, the Binary Cross-Entropy (BCE) loss function is utilized to compute the loss of the prediction outcome relative to the actual label:

$$L(y, \hat{y}) = -\frac{1}{N} \sum_{i=1}^{N} [y_i \cdot \log(\hat{y}_i) + (1 - y_i) \cdot \log(1 - \hat{y}_i)] \tag{8}$$

where $\hat{y}_i$ denotes the predicted value for the i-th drug-protein pair, y_i represents the actual value, and N signifies the total number of DPIs.

3 Materials

Table S1: Detailed statistics of the datasets.

datasets	drugs	proteins	DPIs
BindingDB	3400	886	9166
Davis	68	389	7320
Enzyme	664	445	2969
GPCR	95	223	635
IC	204	210	1476
NR	26	54	90

4 Evaluation Metrics

To impartially assess the performance of each DPI prediction model, we employed metrics including AUC, $AUPR$, *Accuracy* (ACC), *Sensitivity* (SEN), *Precision* (PRE), *Specificity* (SPE), $F1{-}Score$ and Matthews Correlation Coefficient (MCC) in our experiments. The supplementary materials include calculation equations and concise introductions for these metrics. AUC represents the area under the receiver operating characteristic (ROC) curve, illustrating the relationship between the true positive rate (TPR) and the false positive rate (FPR) across different thresholds. $AUPR$, the area under the precision-recall curve, effectively measures a model's classification performance on imbalanced datasets. Below are the calculation equations for each metric:

$$\begin{gathered} ACC = \frac{TP+TN}{TP+TN+FP+FN}, \quad SEN = \frac{TP}{TP+FN}, \quad SPE = \frac{TN}{TN+FP}, \\ PRE = \frac{TP}{TP+FP}, \quad F1-Score = 2 \cdot \frac{PRE \cdot SEN}{PRE+SEN}, \\ MCC = \frac{TP \cdot TN - FP \cdot FN}{\sqrt{(TP+FP)(TP+FN)(TN+FP)(TN+FN)}}, \end{gathered} \tag{9}$$

where TP, TN, FP, and FN denote the counts of correctly identified DPIs, correctly identified non-DPIs, incorrectly identified DPIs, and incorrectly identified non-DPIs, respectively.

Additionally, we provide a brief overview of the application scenarios for these metrics. For instance, the AUC metric evaluates the model's overall classification efficacy on positive and negative samples, whereas $AUPR$ specifically assesses the accuracy of positive sample predictions. ACC reveals the model's overall accuracy rate, whereas SEN, PRE, and SPE measure the accurate identification of positive and negative samples, respectively. The $F1 - Score$, a balanced metric, is calculated as the harmonic mean of precision and recall. MCC, ranging from -1 to +1, is a comprehensive metric considering all classifications (TP, FP, TN, FN) and measures the quality of binary classifications. A value of +1 signifies perfect prediction, 0 indicates random prediction, and -1 denotes a completely inconsistent prediction.

5 Performance Comparison under Isolation Conditions

Furthermore, the model's performance was assessed under isolated conditions, with outcomes detailed in Table S2. "SD" denotes the model's prediction of an interaction between an unknown drug and a known protein, "SP" corresponds to predictions between a known drug and an unknown protein, and "SI" refers to predictions of unknown interactions. Overall, the models performed less effectively in the "SD" and "SP" scenarios compared to the "SI" scenario. As indicated in Table S1, within the BindingDB dataset, drug nodes have an average degree of 2.70, while protein nodes have an average degree of 10.35. Models exhibit inferior performance under the "SD" condition as compared to the "SP" condition, potentially owing to the disparity between sparse drug nodes and denser protein nodes in the dataset. The IGT, Moltrans, TransformerCPI, and IIFDTI models, which utilize transformer technology, demonstrate exemplary performance, affirming the transformer's superior capability in extracting robust node representations. Observations reveal that the proposed model's AUC performance marginally surpasses the second-ranked IIFDTI model in "SD", "SP", and "SI" conditions. Notably, under "SD", "SP", and "SI" conditions, the proposed model's $AUPR$ performance leads by 5.6%, 4.7%, and 3.7% respectively over the runner-up IIFDTI model. This indicates the proposed model's proficiency in not only distinguishing DPIs from non-DPIs but also in uncovering the maximum number of potential DPIs.

Table S2: Results of all models under isolation conditions on BindingDB dataset.

	SD		SP		SI	
method	AUC	AUPR	AUC	AUPR	AUC	AUPR
DeepDTA	0.849±0.005	0.783±0.006	0.854±0.005	0.839±0.007	0.901±0.006	0.829±0.010
GraphDTA	0.853±0.004	0.776±0.005	0.845±0.006	0.818±0.009	0.893±0.007	0.825±0.007
IGT	0.892±0.01	0.867±0.009	0.932±0.009	0.907±0.008	0.937±0.021	0.912±0.011
Moltrans	0.891±0.007	0.833±0.010	0.893±0.006	0.842±0.005	0.925±0.009	0.877±0.008
TransformerCPI	0.919±0.005	0.876±0.015	0.936±0.013	0.908±0.011	0.955±0.013	0.919±0.011
IIFDTI	0.923±0.010	0.871±0.006	0.943±0.005	0.889±0.007	0.951±0.005	0.926±0.005
ours	0.929±0.002	0.927±0.003	0.949±0.003	0.936±0.002	0.966±0.001	0.963±0.002

6 Parameter Experiment

To rigorously assess the influence of each parameter on model performance, we designed a comprehensive suite of parameter-based experiments. The experiments were primarily conducted using the BindingDB, Davis, and Enzyme databases. Owing to their extensive datasets and intricate topological structures, these databases are prevalently utilized in DPI prediction research. During the experiments, we scrutinized the effect of various critical parameters on model efficacy, including the dimensions of the pre-trained model, the count of transformer layers influenced by the global diffusion mechanism, the quantity of attention channels, and the magnitude of the subgraph. Upon concluding the parameter experiments, the results were quantified using metrics such as AUC, $AUPR$, ACC, $F1-score$, and MCC. This approach enables not only the identification of parameters pivotal to model performance but also a thorough comprehension of how various parameter configurations affect model efficacy. The findings from these experiments offer critical insights for enhancing our understanding of the model, refining its structure, and boosting its applicability in drug research and development.

6.1 Impact of Pre-trained Model

In our model, we initially deploy a pre-trained model to assimilate the foundational knowledge of drugs and proteins, which facilitates the extraction of nodes' initial features. Pre-trained models, varying in parameter sizes, exhibit distinct feature extraction capabilities. For drug data, we employ four pre-trained models of varying sizes (with parameters of 450k, 240k, 120k, and 60k) to derive their SMILES features. Regarding proteins, four pre-trained models with parameter sizes of 650M, 150M, 35M, and 8M are utilized to extract amino acid sequence features. Heat maps are employed for visualizing the BindingDB dataset, offering an intuitive display of the features extracted by pre-trained models of various sizes. The outcomes of this visualization are presented in Figure S2.

Additionally, adhering to the principle of controlled variables, we conducted evaluations on the BindingDB dataset. Figure S2 illustrates the model's performance in terms of AUC, $AUPR$, ACC, $F1-score$, and MCC when utilizing pre-trained models of various sizes. Generally, a larger parameter size in the pre-trained model correlates with more precise prediction outcomes. When the drug and protein pre-trained models reach parameter sizes of 60k and 8M, respectively, the differences in feature extraction capabilities among pre-trained models of different sizes become marginal. Under these conditions, the model achieves improved prediction

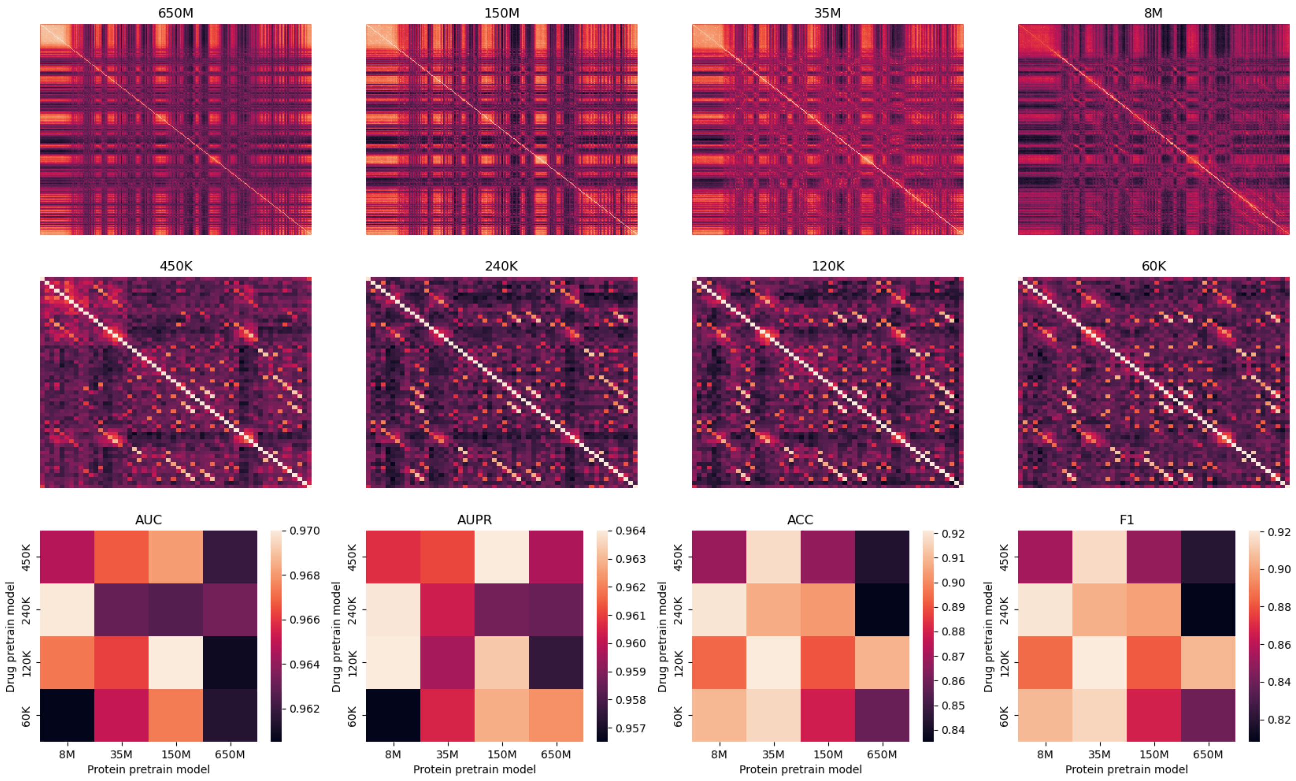

Figure S2: The first row displays drug feature maps, extracted by the PubChem10M_SMILES_BPE pre-trained model of varying sizes. The second row shows protein feature maps, obtained from the esm2_t33_650M_UR50D pre-trained model of diverse sizes. The third row illustrates the performance metrics for models employing pre-trained models with different sizes.

results. Consequently, to conserve computational resources while maintaining accuracy, a smaller pre-trained model can be selected.

6.2 Impact of Subgraph Size

In this study, our model employs two algorithms, MinHash and HyperLogLog, for feature extraction from k-hop subgraphs of nodes. The hyperparameter k is pivotal for determining subgraph size and delineating the node's neighborhood scope. A suite of experiments was executed on three datasets, BindingDB, Davis, and Enzyme, to investigate the influence of varying subgraph sizes on the model's performance. Figure S3 presents the model's prediction outcomes with the k-value set at 1, 2, and 3, respectively.

Analysis of the BindingDB dataset reveals optimal model performance with k set to 1 and 3, while a setting of k at 2 results in the least favorable outcomes. For the Davis dataset, the model performs best when k is at 2 and 3, and least effectively when k is at 1. In the case of the Enzyme dataset, the model's performance remains consistent across different k settings. The results indicate that the model's performance is stable, with minimal variations, despite different subgraph size settings. This suggests that the proposed model exhibits low sensitivity to alterations in the hyperparameter k, allowing for the selection of smaller k values without compromising model performance. This additionally substantiates the notion that message propagation within smaller subgraphs may alleviate the over-smoothing issue commonly seen in GNNs.

6.3 Impact of Transformer Layer Numbers

Our model incorporates a transformer encoder grounded in an energy-constrained diffusion mechanism to discern the subtle interplay between amino acids, facilitating the extraction of nodes' global features. The transformer encoder can be layered sequentially to create a comprehensive global feature extraction module. A sequence of experiments was performed on the BindingDB, Davis, and Enzyme datasets to investigate how varying numbers of transformer layers affect model performance.

Figure S3 delineates the model's predictive accuracy with the transformer encoder layers configured to 1, 2, 3, and 4 layers respectively. Observations from the BindingDB, Davis, and Enzyme datasets indicate that elevating the transformer encoder from 1 to 2 layers optimizes nearly all performance metrics of the model. Evidently, across all datasets, the model's AUC and $AUPR$ metrics are stable with minor variations; in contrast, the ACC,

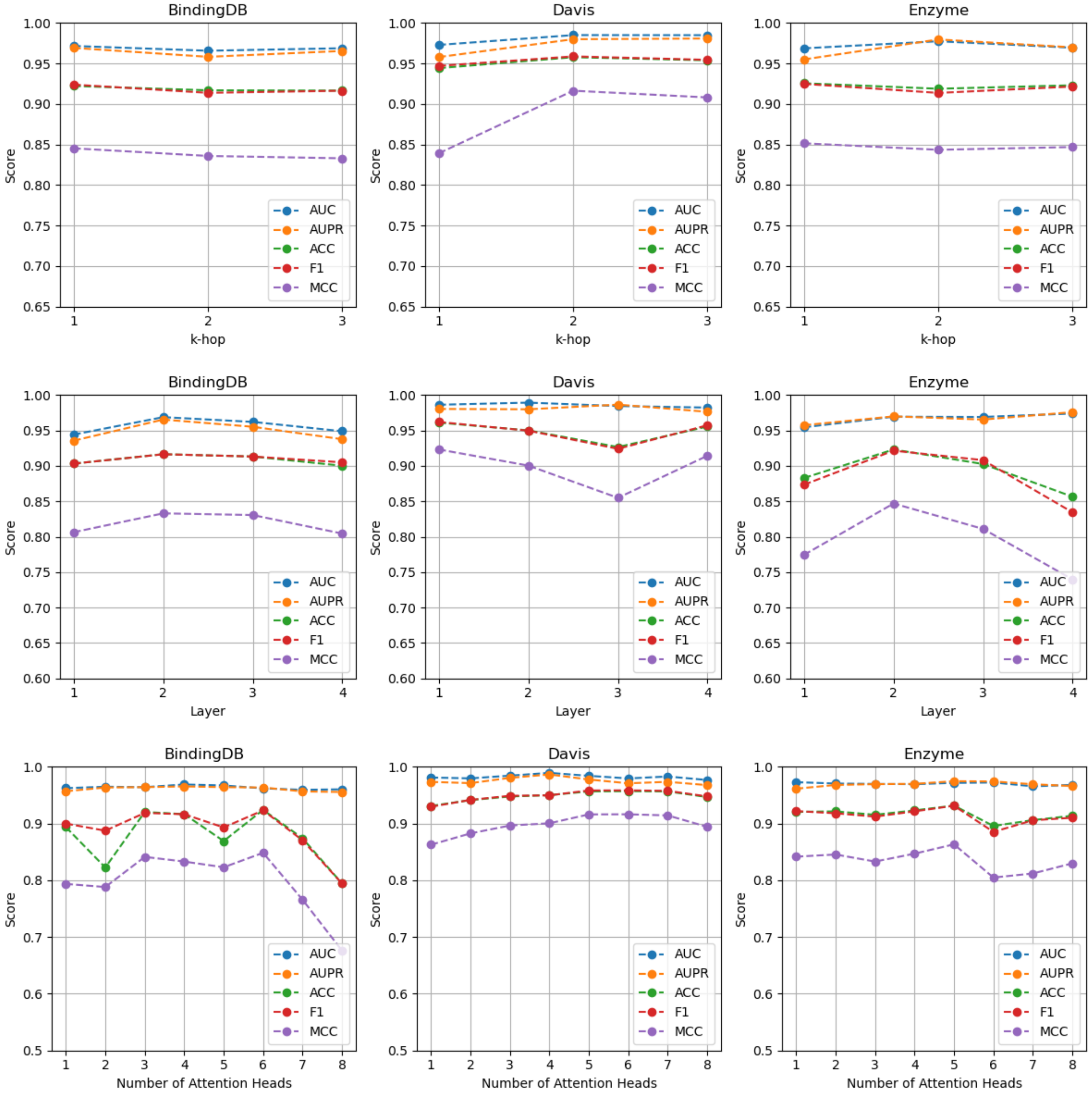

Figure S3: The model's performance is evaluated on varying subgraph sizes, the quantity of transformer encoder layers, and the count of attention heads.

$F1-score$, and MCC metrics display more pronounced and consistent fluctuations. These findings tentatively suggest that a calibrated increase in the number of transformer layers can augment the model's capacity to assimilate complex features. However, an excessive number may lead the model towards overfitting.

6.4 Impact of Transformer Head Numbers

The proposed model's transformer encoder, utilizing a multi-head attention mechanism based on the diffusion process to extract the global feature of nodes. The multi-head attention mechanism outperforms its single-head counterpart by capturing data characteristics across multiple dimensions, thus enriching the model with a plethora of diverse informational perspectives. Evaluations were carried out on three datasets: BindingDB, Davis, and Enzyme, to assess the effects of varying numbers of attention heads on model efficacy. Figure S3 depicts the model's predictive accuracy with the number of transformer attention heads ranging from 1 to 8. The results indicate a slight increase or stabilization in model performance when the number of attention heads is configured between 1 and 5. However, the model's performance on the BindingDB dataset diminishes notably when the number of attention heads exceeds 6. Consequently, it is advisable to configure the number of attention heads within a smaller spectrum to balance performance with computational efficiency.

6.5 Impact of different fusion methods

The proposed model employs direct concatenation to combine proteins' and drugs' final representations, subsequently predicting drug-protein pair scores via a fully connected layer. To assess the impact of various feature fusion strategies on prediction performance, we conducted several additional experiments. In our experiments, we tested feature fusion strategies such as the Hadamard product and summation, yielding the following results.

Table S3: Results of the proposed model using different feature fusion methods on the BindingDB dataset.

fusion methods	AUC	AUPR	ACC	SEN	PRE	SPE	F1-score	MCC
Hadamard product	0.9322	0.9322	0.8079	0.6614	0.9356	0.9544	0.7749	0.6441
summation	0.9368	0.9333	0.7866	0.6057	0.9478	0.9666	0.7398	0.6144
concatenation	0.9688	0.9654	0.9165	0.9157	0.9171	0.9172	0.9164	0.8330

Table S3 demonstrates that simple feature concatenation yielded the highest performance in this study. This suggests that other feature fusion methods in the DPI prediction task might lead to greater information loss. This issue could stem from treating proteins and drugs as heterogeneous nodes. Direct fusion of their features via Hadamard product or summation operations may degrade model performance due to misalignment of features. Conversely, simple feature concatenation obviates the need for strict feature alignment. A "Feature Fusion" section has been included in the supplementary materials to elucidate the effects of different feature fusion techniques on model performance.

7 Discovering New DPIs

In our initial experiments, we focused on the interaction between the drug molecule Calcitriol and a protein complex identified by the PDB number 2MGY [Trump et al., 2004, Jaremko et al., 2014]. Calcitriol represents the final active form of vitamin D3, undergoing several conversion steps in the liver and kidneys. It serves multiple roles in the body, such as enhancing calcium absorption in the small intestine, managing calcium and phosphorus metabolism in bones, and modulating cellular activities in the immune system. 2MGY is a complex formed by the mitochondrial transmembrane transporter and its high-affinity ligand PK11195. The database contains no records of interactions between these two entities. Nonetheless, under isolated conditions, our model predicts a high likelihood of interaction between Calcitriol and 2MGY. Subsequent testing with molecular docking technology revealed the lowest binding energy between Calcitriol and 2MGY to be -4.91, indicating a potential interaction. This confirms that the model, utilizing pre-training and global-local feature extraction strategies, can effectively learn the key structures of drugs and proteins, thereby accurately identifying their interactions. Investigating these newly identified DPIs using the proposed model can deepen our understanding of calcium absorption, its metabolic mechanisms, and guide the treatment of bone-related diseases.

In our second series of molecular docking simulations, we focused on the interaction between a drug identified by PubChem number 118735635 and a protein designated by PDB number 1G3Z [Kim et al., 2023, 2000]. The drug features a complex molecular structure, comprising a carboxylate group, benzene ring, amide group, furan ring, dichlorophenyl group, and pyrazole group, suggesting potential for various biological activities. Protein 1G3Z is identified as carbonic anhydrase II (F131V), a human carbonic anhydrase II mutant crucial

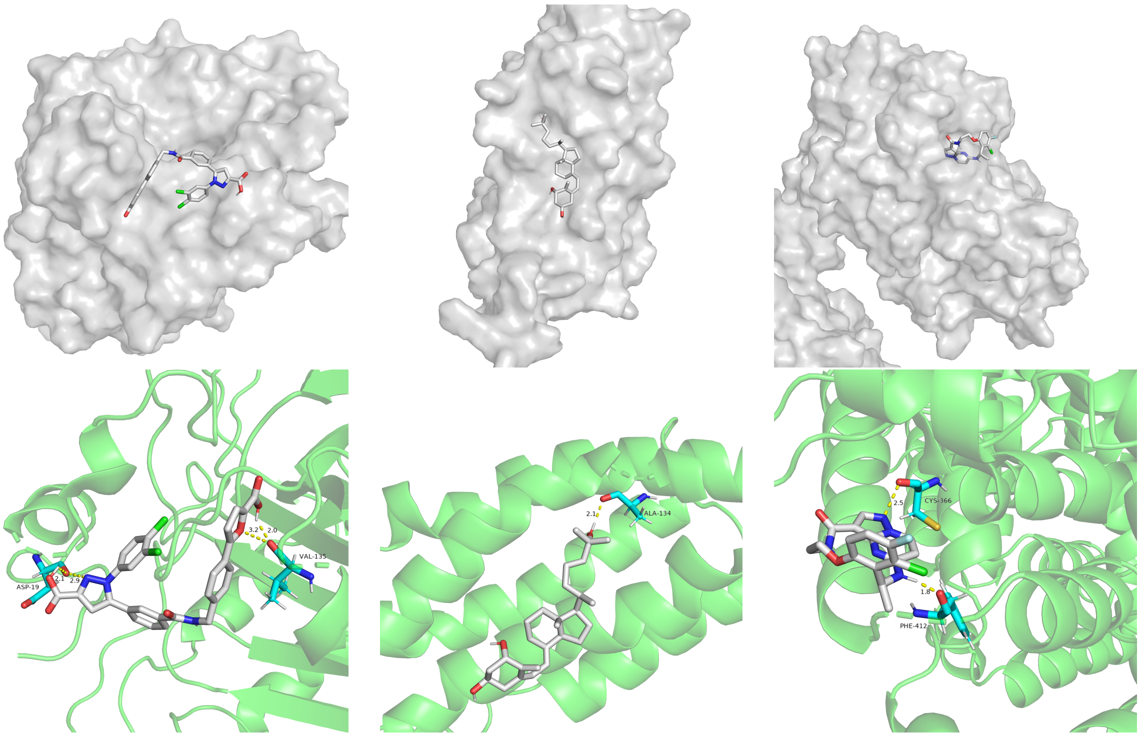

Figure S4: The results of three consecutive groups of molecular docking.

for maintaining acid-base balance and carbon dioxide transport. Investigating such mutations can enhance our understanding of the interplay between protein structure and function, and the influence of specific residues on enzyme activity. Once again, the database lacks any record of this interaction. Nevertheless, the model's predictions indicate a high probability of interaction between the drug and the protein. Subsequent molecular docking analysis revealed that their lowest binding energy reached -5.35. This suggests the potential for these two to combine under natural conditions, thereby supporting the likelihood of their interaction.

In our third series of molecular docking simulations, the interaction between the drug identified by PubChem number 136953041 and the protein complex with PDB number 5EJV was examined [Kim et al., 2023, Enyedy et al., 2016]. The drug molecule comprises pyridine and pyrrole ring structures, carboxamide groups, ether chains, and fluorinated chlorobenzene groups, suggesting its potential in anticancer, antiviral, antibacterial, or other therapeutic applications. Protein 5EJV is characterized as a complex involving $ROR\gamma$, T0901317, and EBI96, where $ROR\gamma$ functions as an orphan nuclear receptor. As a transcriptional molecule, it has the capacity to regulate the expression of various genes. T0901317 can influence $ROR\gamma$'s activity, consequently impacting the genes it controls. EBI96, a coactivator peptide, binds to nuclear receptors, amplifying their transcriptional activities. Investigating this complex is vital for comprehending $ROR\gamma$'s mechanism of action in vivo and holds potential significance in developing therapeutics targeting $ROR\gamma$. Once more, this interaction is not recorded in the database. The model's predictions indicate a high likelihood of interaction between these entities. Molecular docking results revealed that their lowest binding energy was -5.4. This indicates the potential for interactions between them.

These results validate the accuracy and reliability of our model and suggest its capability to identify and explore unknown drug targets, offering novel insights in drug research and development.

References

I. J. Enyedy, N. A. Powell, J. Caravella, K. van Vloten, J. Chao, D. Banerjee, D. Marcotte, L. Silvian, A. McKenzie, V. S. Hong, et al. Discovery of biaryls as rorγ inverse agonists by using structure-based design. *Bioorganic & Medicinal Chemistry Letters*, 26(10):2459–2463, 2016.

Ł. Jaremko, M. Jaremko, K. Giller, S. Becker, and M. Zweckstetter. Structure of the mitochondrial translocator protein in complex with a diagnostic ligand. *Science*, 343(6177):1363–1366, 2014.

C.-Y. Kim, J. S. Chang, J. B. Doyon, T. T. Baird, C. A. Fierke, A. Jain, and D. W. Christianson. Contribution of fluorine to protein- ligand affinity in the binding of fluoroaromatic inhibitors to carbonic anhydrase ii. *Journal of the American Chemical Society*, 122(49):12125–12134, 2000.

S. Kim, J. Chen, T. Cheng, A. Gindulyte, J. He, S. He, Q. Li, B. A. Shoemaker, P. A. Thiessen, B. Yu, et al. Pubchem 2023 update. *Nucleic acids research*, 51(D1):D1373–D1380, 2023.

D. L. Trump, P. A. Hershberger, R. J. Bernardi, S. Ahmed, J. Muindi, M. Fakih, W.-D. Yu, and C. S. Johnson. Anti-tumor activity of calcitriol: pre-clinical and clinical studies. *The Journal of steroid biochemistry and molecular biology*, 89:519–526, 2004.
